# Sensing Performance and Efficiency of Two Energy Transfer-Based Two-Photon Fluorescent Probes for pH

**DOI:** 10.3390/s18124407

**Published:** 2018-12-13

**Authors:** Yujin Zhang, Wei Hu

**Affiliations:** 1 School of Electronic and Information Engineering (Department of Physics), Qilu University of Technology (Shandong Academy of Sciences), Jinan 250353, China; zhangyujin@qlu.edu.cn; 2 School of Chemistry and Pharmaceutical Engineering, Qilu University of Technology (Shandong Academy of Sciences), Jinan 250353, China; 3 Hefei National Laboratory for Physical Sciences at the Microscale, iChEM (Collaborative Innovation Center of Chemistry for Energy Materials), School of Chemistry and Materials Science, University of Science and Technology of China, Hefei 230026, China

**Keywords:** fluorescent probe, two-photon absorption, pH sensor, time-dependent density functional theory

## Abstract

The design and synthesis of fluorescent probes for monitoring pH values inside living cells have attracted great attention, due to the important role pH plays in many biological processes. In this study, the optical properties of two different two-photon fluorescent probes for pH are studied. The ratiometric sensing of the probes are theoretically illustrated. Meanwhile, the recognitional mechanisms of the probes are investigated, which shows the energy transfer process when react with H^+^. Specially, the calculated results demonstrate that Probe1 possesses a higher energy transfer efficiency and a larger two-photon absorption cross-section than Probe2, indicating it to be a preferable pH fluorescent probe. Therefore, the influence of connection between the donor and the acceptor on the sensing performances of the probe is demonstrated. Our results help to understand the experimental observations and provide a theoretical basis to synthesize efficient two-photon fluorescent probes for monitoring pH changes.

## 1. Introduction

Intracellular pH value plays a pivotal role in biological processes, such as the growth of the cell, transportation of the ions, shrinkage of muscle, and activity of the enzyme [1,2]. Abnormal pH values can lead to cellular dysfunctions and cause serious diseases, such as Alzheimer’s, cancer, arthritis, and many others [3,4,5]. Therefore, monitoring pH changes inside living cells is of great importance to research on the pathological and physiological processes of the cells [6,7].

The value of pH can be assessed by various techniques, including nuclear magnetic resonance, permeable microelectrodes, absorbance spectroscopy, and fluorescence microscopy. Thereinto, fluorescence microscopy with pH-sensitive probes has become an indispensable tool, due to its high sensitivity and selectivity, simple operation, and non-destructive use in living cells [8,9]. In the past decades, many fluorescent sensors for pH have been reported. However, most of the reported pH probes are based on one-photon excitation, in which the light penetration and imaging resolution are limited [10,11,12,13]. In this context, two-photon fluorescence microscopy, using two photons with lower energy as the excitation source, has attracted much attention for its evident advantages, such as localized excitation, increased penetration depth, and low tissue autofluorescence and self-absorption [14,15,16].

Up to now, a variety of two-photon fluorescent probes have been developed for measuring pH. For instance, Kim et al. reported a new family of small molecule and ratiometric two-photon probes derived from benzimidazole for monitoring acidic pH values based on the intramolecular charge transfer (ICT) mechanism [17]. Besides, a novel tumor-targeting and lysosome-specific two-photon fluorescent probe for imaging pH changes by photoinduced electron transfer (PET) mechanism was synthesized [18]. Usually, these probes are designed on basis of single-emission intensity changes and can be easily affected by the environmental conditions, instrumental efficiency, and probes’ concentration. An effective approach to eliminate these interferences is to use a ratiometric fluorescent probe, which adopts a built-in correction of two emission bands [19]. Several strategies, including Förster resonance energy transfer (FRET) and through bond energy transfer (TBET), have been employed to design ratiometric fluorescent probes. For FRET-based fluorescent probes, the energy donor and acceptor are linked by a flexible and electronically non-conjugated spacer, and there is a large overlap between the emission of the donor and the absorption of the acceptor. As a result, when the donor moiety is excited, the energy transfer process occurs through space, and the emission of acceptor is observed. In the case of TBET-based fluorescent probes, the linker between the energy donor and the acceptor is a rigid and electronically conjugated bond. Energy transfer can, thus, occur through the bond directly, and spectral overlap is not necessary.

Many FRET/TBET-based fluorescent probes have been put forward for the ratiometric detection of pH so far [20,21,22]. Very recently, Zhou et al. designed a unique type of ratiometric TBET-based two-photon fluorescent probe (hereafter named as Probe1), in which a two-photon fluorophore (naphthalimide derivative) and a rhodamine B fluorophore were directly connected, for imaging of lysosomal pH in living cells and tissues [23]. By adopting the same donor and acceptor of Probe1 and connecting them with a flexible piperidine linker, they reported a FRET-based two-photon fluorescent pH probe (hereafter named as Probe2), which showed high imaging resolution and deep tissue imaging depth [24]. Although the experimental measurements demonstrate that both Probe1 and Probe2 are reliable and specific probes for pH, there are few theoretical investigations on the underlying mechanism of these probes. Furthermore, for these energy transfer-based probes, the rate of energy transfer between the donor and acceptor is a very important indicator for evaluating the efficiency of the probe. A uniform standard to evaluate the energy transfer rate experimentally is lacking. Thus, comparison on the energy transfer rate of the probes, at the same theoretical level, is greatly desired. In this work, we carry out theoretical studies on the optical properties of Probe1 and Probe2 in the absence and presence of H^+^. Special attention has been paid to analyzing the recognitional mechanism of the probes using a molecular orbital diagram. Importantly, a feasible approach has been used to predict the energy transfer rate between the energy donor and acceptor of the probe. Our theoretical investigations can provide helpful information for rationally designing fluorescent probes with high efficiency.

## 2. Computational Methods

In this work, geometrical structures of all the studied molecules are fully optimized by using the density functional theory with the 6-31G(d, p) basis set, and the B3LYP hybrid functional [25]. Frequency analyses are carried out to ensure no imaginary frequency is obtained. On the basis of the optimized ground state structures, the one-photon absorption (OPA) spectra are calculated by the time-dependent density functional theory (TD-DFT) at the same calculation level, and the geometry optimization of the first excited state and emission spectra are obtained by B3LYP/6-31G(d, p). All the above calculations are implemented with the Gaussian09 program package [26]. Considering that all the experimental measurements are carried out in an aqueous environment [23,24], the effect of water solution is taken into account within the self-consistent reaction field theory by using the polarizable continuum model (PCM) [27]. It should be noted that upon the addition of analyte, the analyte concentration is increased, and the free probes are transformed gradually. In this process, the solution includes both the free probe and the product until all the probe molecules are reacted. Thus, the actual fluorescent probe changes its absorbance and fluorescent intensity with an increase in analyte concentration. In the calculation, we investigate the properties of the free probe and the product, directly, to compare with the experimental measurements.

The transition probability of one-photon absorption and emission can be described by the oscillator strength

(1) $$\delta_{OPA(OPE)}=\frac{2\omega_{ij}}{3}\sum_{\alpha }|\langle i|\mu_{\alpha }|j\rangle {|}^{2},$$

where *ω_ij_* denotes the energy difference between the states *i* and *j*, *μ_α_* is the electric dipole moment operator, and the summation is performed over the axes α={x,y,z}.

Moreover, the DALTON2013 program [28] is employed to calculate the two-photon absorption (TPA) properties of the molecules in an aqueous environment with the B3LYP functional and 6-31G(d, p) basis set, using response theory. The solvent effect is taken into account within the PCM. The radiuses of cavities are taken from the Gaussian calculations.

From the sum-over-state formulas, the resonant two-photon absorption matrix element can be expressed as [29]

(2) $$S_{\alpha \beta }=\sum_{s}\left[\frac{\langle i|\mu_{\alpha }|s\rangle \langle s|\mu_{\beta }|j\rangle }{\omega_{si}-\omega }+\frac{\langle i|\mu_{\beta }|s\rangle \langle s|\mu_{\alpha }|j\rangle }{\omega_{si}-\omega }\right],$$

where *μ*_*α*(*β*)_ denotes the dipole moment operator in the direction *α*, *β*є(*x*,*y*,*z*); ***ω*** is the energy of the incident laser beam and equal to half of the excitation energy of the final state *j*; and the summation covers all the intermediate states. The TPA cross-section of a molecule is given by orientational averaging over the TPA probability:

(3) $$\delta_{TPA}=\sum_{\alpha ,\beta }\left[F\times S_{\alpha \alpha }S_{\beta \beta }^{*}+G\times S_{\alpha \beta }S_{\alpha \beta }^{*}+H\times S_{\alpha \beta }S_{\beta \alpha }^{*}\right].$$

Here, the coefficients *F*, *G*, and *H* are related to the polarization of the excitation laser pulse. For linearly polarized light, the values of *F*, *G*, and *H* are 2, 2, and 2; and for the circular case, the values are—2, 3, and 3, respectively. The summation covers the molecular *x*, *y*, and *z* axes, namely, *α*, *β*є(*x*,*y*,*z*).

The TPA cross-section, directly comparable with experiment, is then defined as [30]

(4) $$\sigma_{TPA}=\frac{4\pi^{2}{a_{0}}^{5}\alpha \omega^{2}g(\omega )}{15c\Gamma }\delta_{TPA}.$$

Here, *a*_0_, *α*, and *c* are the Bohr radius, the fine structure constant, and the speed of the light, respectively; *ħω* is the incident photon energy; *g*(*ω*) provides the spectral line profile; and the lifetime broadening of the final state Γ is assumed to be a typical value of 0.1 eV.

## 3. Results and Discussion

### 3.1. Molecular Structure

**Figure 1:**
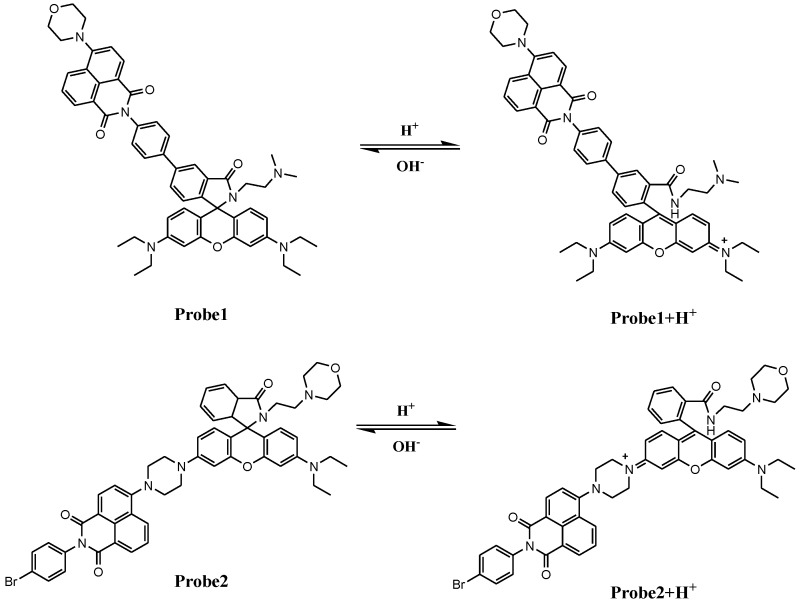
Molecular structures of the studied molecules.

**Figure 2:**
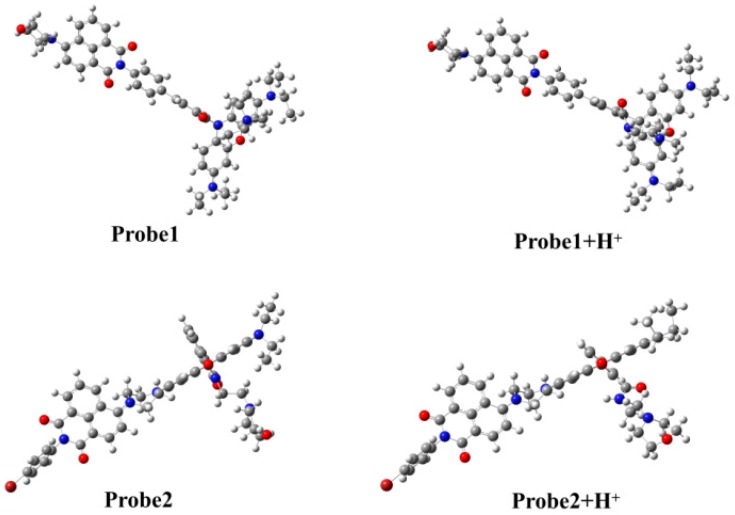
The optimized ground state molecular structures of the studied molecules.

The structures of all the studied molecules in the present work are shown in Figure 1. One can see that both Probe1 and Probe2 have the same donor (naphthalimide derivative) and acceptor (rhodamine B unit). Nevertheless, the connection between the two parts is different. When reacting with H^+^, the ring-closed rhodamine in the probes are induced to be ring-opened forms, as shown in Probe1+H^+^ and Probe2+H^+^. The optimized ground state geometries of the molecules in H_2_O show that the donor and acceptor moieties of the probes, in the absence and presence of H^+^, are coplanar, and there is a large tortuosity between them (see Figure 2), which is beneficial to the energy transfer process from donor to acceptor.

### 3.2. One-Photon Absorption

**Figure 3:**
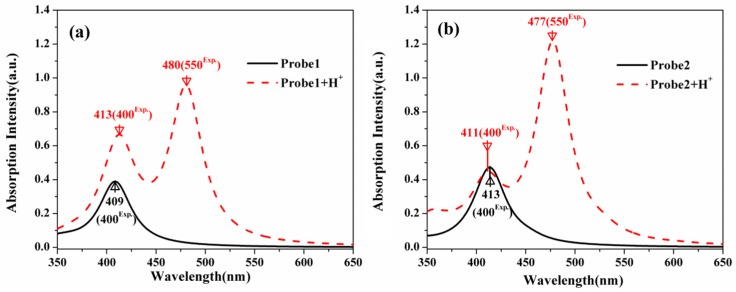
The one-photon absorption (OPA) spectra of (**a**) Probe1, Probe1+H^+^ and (**b**) Probe2, Probe2+H^+^.

The OPA process is closely related to the fluorescence of the molecules, thus, it is necessary to analyze the molecular OPA properties. The absorption spectra of the studied molecules in H_2_O are presented in Figure 3. It can be seen that both Probe1 and Probe2 have one absorption peak, located at 409 nm and 413 nm, respectively. With the presence of H^+^, the spectral shape of Probe1+H^+^ and Probe2+H^+^ have pattern characterized by two absorption bands with different intensities. The absorption peaks of Probe1+H^+^ and Probe2+H^+^, with weaker intensities located at about 410 nm, are nearly the same as those of Probe1 and Probe2. On the other hand, the newly appeared peaks with larger intensities at about 480 nm for Probe1+H^+^ and Probe2+H^+^ can be attributed to the open-ring rhodamine (see analysis of the molecular orbitals involved hereinafter).

**Figure 4:**
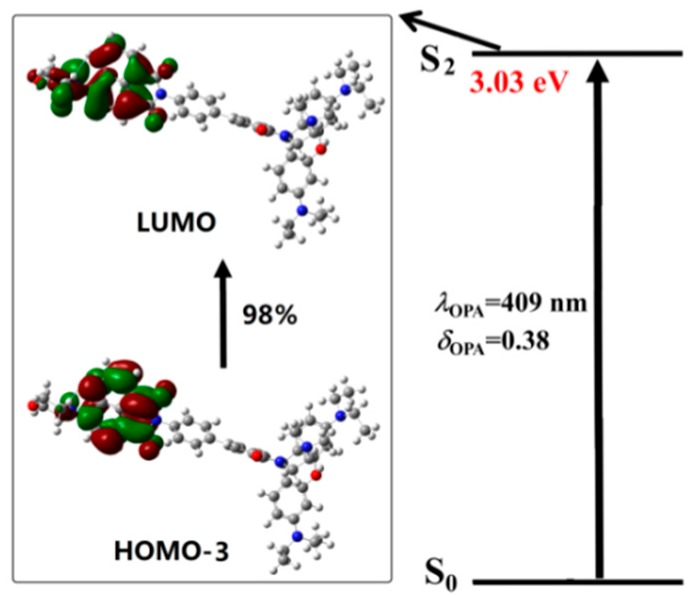
The transition corresponding to the OPA peak of Probe1.

**Figure 5:**
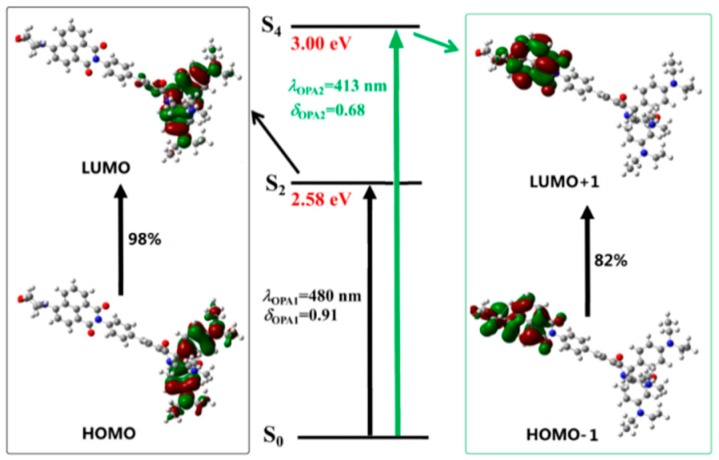
The transition corresponding to the OPA peak of Probe1+H^+^.

**Figure 6:**
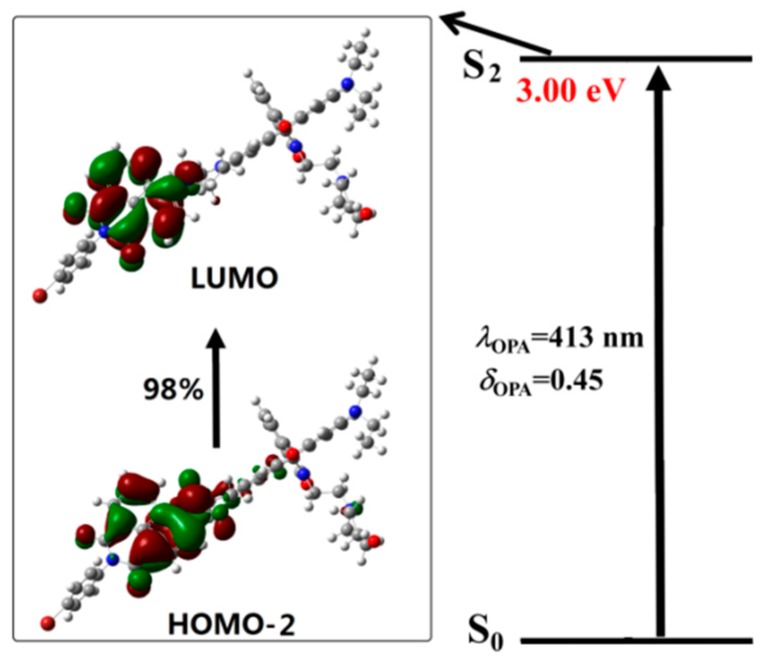
The transition corresponding to the OPA peak of Probe2.

**Figure 7:**
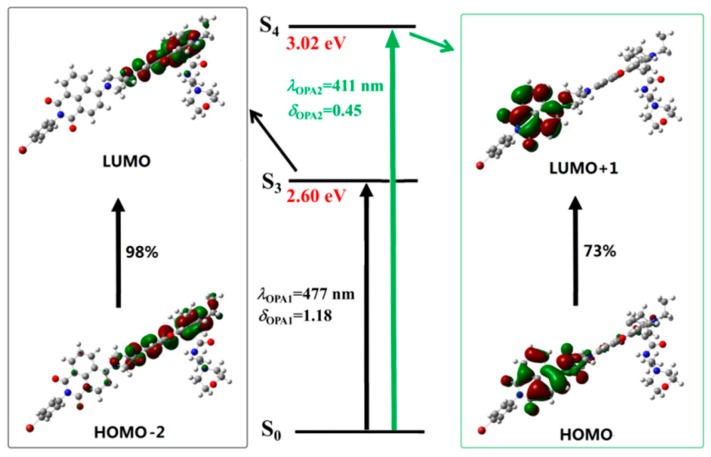
The transition corresponding to the OPA peak of Probe2+H^+^.

To get a better understanding of the spectral phenomenon, the details of the transition corresponding to the OPA peaks of all the studied molecules are shown in Figure 4, Figure 5, Figure 6 and Figure 7. As shown in Figure 4, the OPA peak of Probe1 originates from the HOMO−3 to LUMO transition (here, the HOMO and LUMO represent the highest occupied molecular orbital and the lowest unoccupied molecular orbital, respectively). It can be observed that the absorption peak of Probe1 is distributed on the donor moiety, and the corresponding transition is the ground state S_0_ to the second excited state S_2_. For Probe1+H^+^ (see Figure 5), the long wavelength absorption peak is contributed to by the HOMO to LUMO transition, and localized on the acceptor moiety, corresponding to the S_0_ to S_2_ transition. And the short wavelength absorption peak of Probe1+H^+^ results from the HOMO−1 to LUMO+1 transition, which is localized on the molecular donor. Similar changing trends occur in Probe2 and Probe2+H^+^ (see Figure 6 and Figure 7), revealing that there is no strong electronic interaction between the donor and acceptor. Thus, the donor and acceptor can be individually excited at their characteristic absorption wavelength, which is conductive to the energy transfer process.

### 3.3. Fluorescent Emission

**Figure 8:**
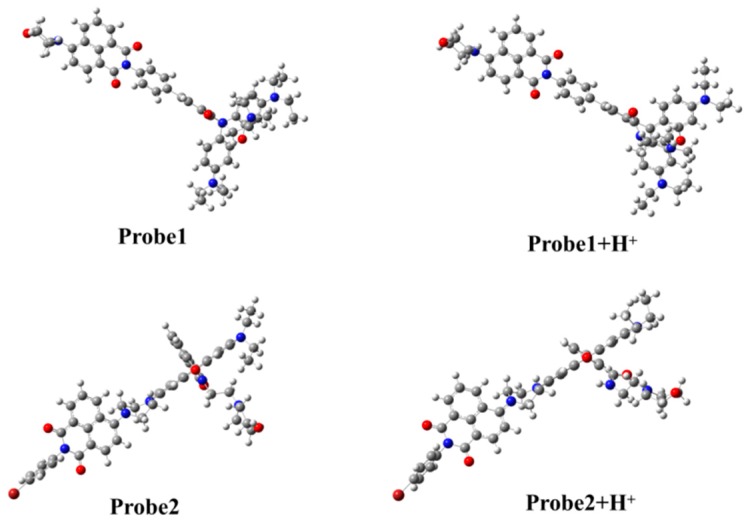
The optimized first excited state molecular structures of the studied molecules.

Discernible changes on the fluorescent signal either on the wavelength or the intensity should be shown when a probe reacts with the analyte. The fluorescence properties of Probe1 and Probe2 in the absence and presence of H^+^ are calculated by optimizing the first excited state geometries of the molecules. The optimized first excited state molecular structures of the studied molecules are given in Figure 8. It can be found that the geometries show little change when excited from the ground state to the first excited state.

**Figure 9:**
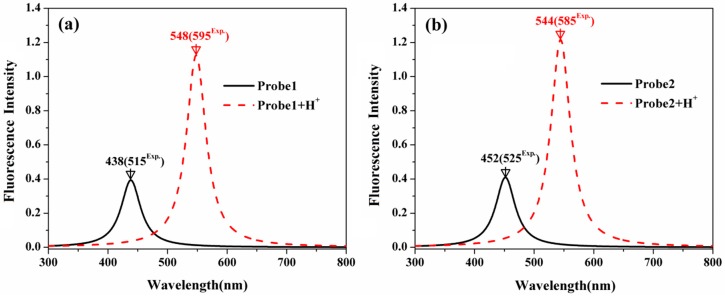
The OPE spectra of (**a**) Probe1, Probe1+H^+^ and (**b**) Probe2, Probe2+H^+^.

Figure 9 shows the fluorescent spectra of the studied molecules. In comparison with the emission wavelength of Probe1 at 438 nm, that of Probe2 is redshifted to 452 nm, which agrees with the trend in the experiments [23,24]. With the addition of H^+^, the fluorescent wavelengths of the probes exhibit large redshifts, and the fluorescent intensities are strongly enhanced. Moreover, the H^+^-induced redshift is more significant for Probe1 than Probe2. Namely, the redshifts on the fluorescence are 110 nm and 92 nm for Probe1 and Probe2 when reacting with H^+^, respectively, which is in reasonable agreement with the experimental measurements of 80 nm and 60 nm [23,24]. Notable, although the calculated values correspond with the experimental results on the trend, there are still numerical discrepancies. This may result from the vibrational contributions and the interaction between the solute and solvent, that have not been considered in the calculations.

**Figure 10:**
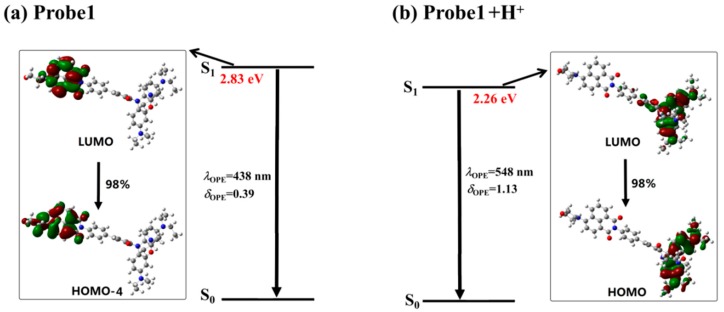
The transition corresponding to the OPE peak of (**a**) Probe1 and (**b**) Probe1+H^+^.

**Figure 11:**
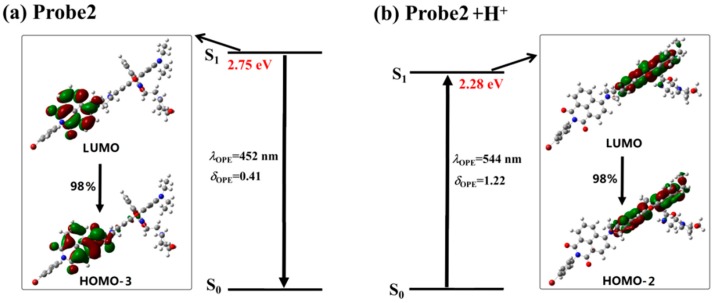
The transition corresponding to the OPE peak of (**a**) Probe2 and (**b**) Probe2+H^+^.

In order to analyze the emission process, the molecular orbitals involved in the transitions corresponding to the OPE peaks of all the studied molecules are demonstrated in Figure 10 and Figure 11. It can be seen that the emission processes of the molecules are mainly contributed by the transitions from the first excited state to the ground state, which conforms to the Kasha’s rule. In addition, the emission is localized on the donor part for the free probes, whereas it is on the acceptor moiety when the probes react with the H^+^. Thus, the fluorescence of the probes features the emission wavelength of the naphthalimide derivative and the rhodamine B unit in the absence and presence of H^+^, respectively. Consequently, the fluorescence shows great redshifts when the probes react with H^+^, which agrees with the experimental observations.

In addition, compared with the Stokes shifts of 1619 cm^−1^ for Probe1, that of Probe2 is increased to 2089 cm^−^^1^. After reacting with H^+^, the largest Stokes shifts for Probe1+H^+^ and Probe2+H^+^ are 2584 cm^−^^1^ and 2582 cm^−^^1^, respectively. Thus, one can predict that the interference from the absorption on the fluorescence for Probe1 and Probe2 can be receded under acidic conditions. Moreover, the Stokes shift of Probe1 increased from 1619 to 2584 cm^−^^1^ in the presence of H^+^, which is larger than that of Probe2 from 2089 to 2582 cm^−^^1^, indicating Probe1 to be a probe with less interference.

### 3.4. Responsive Mechanism

Although the recognitional mechanisms of Probe1 and Probe2 are referred to in the experiments [23,24], theoretical rationalization on this issue has not been discussed up to now. In order to gain further insight into the underlying mechanism of the probes, the response process of the probes to the excitation light are specifically exhibited by using the molecular orbital distribution diagrams. The absorption process of Probe1 in Figure 4 shows that when excited by the light, the mainly allowed electronic transition is localized on the donor moiety. Then, the molecule at a higher excited state will relax to the lowest vibrational level of the first excited state, followed by decaying back to the ground state with the photon emission according to Kasha’s rule. From the distributions of the molecular orbitals involved in the emission process (see Figure 10), one can clearly see that the fluorescence of Probe1 is also distributed on the donor moiety.

When reacting with H^+^, the situation is greatly changed. As shown in Figure 5, upon excitation with the laser wavelength of 413 nm, the molecule Probe1+H^+^ is excited to the fourth excited state through the transition localized on the naphthalimide derivative. However, Figure 10 shows that the emission of Probe1+H^+^ is localized on the accepter unit. Thus, the fluorescence from the open-ring rhodamine will be observed. This process indicates that the energy transfer occurs between the donor and acceptor of Probe1+H^+^. Consequently, the energy transfer off–on transform, stimulated by the addition of H^+^, induces fluorescent signal from the donor to the acceptor.

The photoabsorption and photoemission processes of Probe2 and Probe2+H^+^ are similar to those of Probe1 and Probe1+H^+^. That is to say, both the absorption and emission of Probe2 are localized on the donor moiety. However, Probe2+H^+^ can be stimulated by exciting the molecular donor while the radiative transition is localized on the molecular acceptor. In general, the energy transfer process in Probe2+H^+^ is intuitively demonstrated.

To further assign the recognitional mechanisms to FRET or TBET, the connection between the donor and acceptor should be considered. As we have mentioned above, for probes based on the FRET and TBET strategies, their donor and acceptor are linked by flexible non-conjugated spacer and rigid conjugated spacer, respectively. As a result, Probe1 is designed as a TBET-based probe, while Probe2 is a FRET-based probe.

### 3.5. Energy Transfer Rate

For an energy transfer-based system, the energy transfer rate is a very important parameter, which directly describes the efficiency of the probe. Here, we adopt a feasible method to calculate the energy transfer rate, i.e., the energy transfer probability per unit time, by applying the Fermi’s golden rule. The detailed theory has been reported in [31].

**Figure 12:**
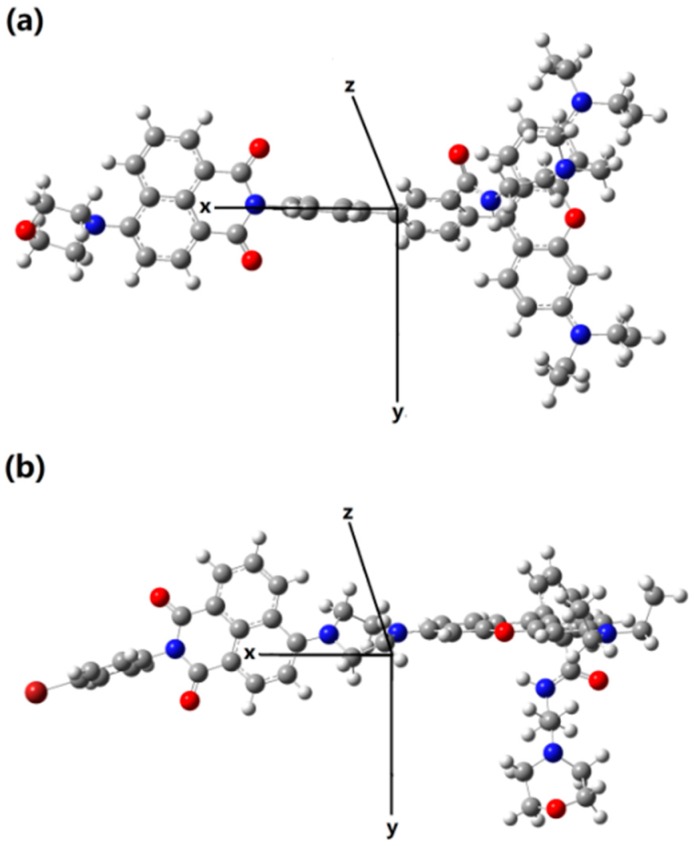
Schematic of coordinate direction for (**a**) Probe1+H^+^ and (**b**) Probe2+H^+^.

In this section, the single molecule is divided to two parts, i.e., the naphthalamide (the donor) and the rhodamine B (the acceptor). Due to the fact that the energy transfer process actually means the acceptor part is excited by absorbing the fluorescence of donor, we thus calculate the emission of the donor moiety and the absorption of the acceptor moiety. In this paper, the short axis of the xanthene structure of rhodamine, the long axis of the xanthene, and the direction perpendicular to the plane of xanthene are set to be the *x*-axis, *y*-axis, and *z*-axis for Probe1+H^+^, while they are set to be the *z*-axis, *x*-axis, and *y*-axis for Probe2+H^+^ (see Figure 12). On basis of the fixed coordinates, the transition wavelength and strength, corresponding to both the emission of donor and the absorption of the acceptor, are calculated and listed in Table 1. Obviously, the overlap between the emission wavelengths of the donor and the absorption wavelength of the acceptor for Probe2+H^+^ are much larger than that for Probe1+H^+^. This confirms the recognitional mechanisms of Probe1+H^+^ and Probe2+H^+^ to be TBET and FRET, respectively.

As is known, the energy transfer rate depends crucially on the distance vector between the donor and acceptor of the molecule [32]. Data in Table 1 show that the distance has the largest component in the *x*-axis direction, both for Probe1+H^+^ and Probe2+H^+^. Although the total distances between the donor and acceptor are almost the same for the two molecules, the energy transfer rate of Probe1+H^+^ is about 2.7 times larger than that of Probe2+H^+^, indicating that Probe1 can be a preferable probe with higher efficiency.

### 3.6. Two-Photon Absorption

The analyses on the fluorescence and recognitional mechanism of the probes have demonstrated that Probe1 and Probe2 can effectively identify H^+^. Importantly, the naphthalimide derivative is reported as a good two-photon fluorophore. Hence, the TPA properties of the studied molecules are theoretically discussed. In the range of 700–900 nm, the two-photon excitation energy, the corresponding TPA wavelength, and the TPA cross-section are summarized in Table 2. One can see from Table 2 that the free probes do not have significant two-photon response in the wavelength range of 700–900 nm (*σ*_TPA_ < 50 GM). In the presence of H^+^, the maximum TPA cross-sections of Probe1+H^+^ and Probe2+H^+^ exhibit great enhancement from 47 to 300 GM, and from 27 to 117 GM, respectively, which is contributed to by the open-ring rhodamine. Note that the maximum TPA cross-section of Probe1 is much larger than that of Probe2, both in the absence and presence of H^+^. Due to the probes possessing similar donor and acceptor, but different connections, it can be concluded that the connection between the donor and acceptor plays a dominant role in the TPA performance. As a consequence, Probe1 is proven to be a preferable candidate as the two-photon fluorescent probe for H^+^ in comparison with Probe2.

For Probe1, using a laser with 826 nm as the excitation source, the probe is excited and the fluorescence emitted from the donor part can be observed on account of no energy transfer. When H^+^ is present, the energy transfer process will occur in Probe1+H^+^, and the fluorescence from the acceptor can be observed. In this case, the detection of H^+^ is efficiently achieved, with weaker photodamage and photobleaching.

## 4. Conclusions

In this work, the optical properties, recognitional mechanisms, and energy transfer rate of two fluorescent chemosensors for pH are theoretically investigated. The calculated results show that the photoabsorption and photoemission of the probes are significantly changed with the addition of H^+^, indicating the probes can serve as efficient fluorescent probes for ratiometric pH measurements. Molecular orbital diagrams are utilized to elucidate the sensing mechanisms of the probes, where the TBET and FRET processes have been specifically modeled. Importantly, the energy transfer rates of the energy transfer-based fluorescent probes are compared based on the same theory level, and the results reveal that Probe1 has a higher energy transfer rate, suggesting that Probe1 can be a promising TBET-based fluorescent sensor compared with Probe2. In addition, two-photon absorption cross-section is largely enhanced when the probes react with H^+^. It shows that both probes are efficient two-photon fluorescent probes. Specially, the two-photon cross-section of Probe1 is larger both in the absence and in the presence of H^+^, which confirms Probe1 to be a better H^+^ chemosensor. Further, the effect of the connection between the donor and the acceptor of the probe is demonstrated. Our theoretical investigations revealed the underlying mechanisms that satisfactorily explained the experimental results, providing efficient information on designing more two-photon fluorescent probes.

## Figures and Tables

**Table 1:** The transition wavelength λ (nm) and the corresponding transition strength *δ* (a.u.) of the emission of the donor (Donor-emission) and the absorption of the acceptor (Acceptor-absorption), the distance vector R*_x_*_,*y*,*z*_ (Å), and energy transfer rate *K_DA_* (10^4^) between the donor and acceptor for Probe1+H^+^ and Probe2+H^+^ in H_2_O.

Molecule	Moiety	λ	*δ*	Distance Vector				*K_DA_*
				R*_x_*	R*_y_*	R*_z_*	R	
Probe1+H^+^	Donor-emission	378	0.48	12.86	−0.78	0.43	12.89	5.2
	Acceptor-absorption	479	0.94					
Probe2+H^+^	Donor-emission	423	0.24	12.07	0.63	1.63	12.20	1.9

**Table 2:** The two-photon excitation energy *E_TPA_* (eV), the corresponding TPA wavelength λ*_TPA_* (nm), and the TPA cross-section σ*_TPA_* (GM = 10^−50^ cm^4^ s/photon) for the studied molecules in H_2_O.

Molecule	*E_TPA_*	λ*_TPA_*	σ*_TPA_*	Molecule	*E_TPA_*	λ*_TPA_*	σ*_TPA_*
Probe1	3.03	818	47	Probe1+H^+^	2.90	852	252
	3.08	802	0		3.01	821	42
	3.22	768	0		3.05	810	300
	3.40	727	0		3.26	758	8
	3.50	706	43		3.37	733	0
	3.53	700	0		3.39	729	35
Probe2	2.77	892	20	Probe2+H^+^	3.02	822	117
	3.00	826	27		3.12	792	32
	3.04	813	0		3.13	790	1
	3.32	744	0		3.15	785	15
	3.49	708	0		3.23	765	13
